# Extracellular vesicles from bone marrow-derived mesenchymal stromal cells support *ex vivo* survival of human antibody secreting cells

**DOI:** 10.1080/20013078.2018.1463778

**Published:** 2018-04-26

**Authors:** Doan C. Nguyen, Holly C. Lewis, Chester Joyner, Vivien Warren, Haopeng Xiao, Haydn T. Kissick, Ronghu Wu, Jacques Galipeau, F. Eun-Hyung Lee

**Affiliations:** a Division of Pulmonary Allergy, Critical Care, & Sleep Medicine, Emory University, Atlanta, GA, USA; b Departments of Pediatrics and Hematology & Medical Oncology, Emory University School of Medicine, Atlanta, GA, USA; c International Center for Malaria Research, Education and Development, Emory Vaccine Center, Yerkes National Primate Research Center, Emory University, Atlanta, GA, USA; d School of Chemistry and Biochemistry, Georgia Institute of Technology, Atlanta, GA, USA; e Emory Vaccine Center and Department of Urology, Emory University, Atlanta, GA, USA; f Department of Medicine and University of Wisconsin Carbone Cancer Center, University of Wisconsin in Madison, Madison, WI, USA

**Keywords:** Mesenchymal stromal cell, extracellular vesicles, antibody secretion cell, plasma cell

## Abstract

Extracellular vesicles (EVs) from bone marrow (BM)-derived mesenchymal stromal cells (BM-MSC) are novel mechanisms of cell-cell communication over short and long distances. BM-MSC have been shown to support human antibody secreting cells (ASC) survival *ex vivo*, but whether the crosstalk between the MSC-ASC interaction can occur via EVs is not known. Thus, we evaluated the role of EVs in ASC survival and IgG secretion. EVs were isolated from irradiated and non-irradiated primary BM-MSC and were quantified. They were further characterized by electron microscopy (EM) and CD63 and CD81 immuno-gold EM staining. Human ASC were isolated via fluorescence-activated cell sorting (FACS) and cultured *ex vivo* with the EV fractions, the EV-reduced fractions, or conventional media. IgG Elispots were used to measure the survival and functionality of the ASC. Contents of the EV fractions were evaluated by proteomics. We saw that both irradiated and non-irradiated MSC secretome preparations afforded vesicles of a size consistent with EVs. Both preparations appeared comparable in EM morphology and CD63 and CD81 immuno-gold EM. Both irradiated and non-irradiated EV fractions supported ASC function, at 88% and 90%, respectively, by day 3. In contrast, conventional media maintained only 4% ASC survival by day 3. To identify the specific factors that provided *in vitro* ASC support, we compared proteomes of the irradiated and non-irradiated EV fractions with conventional media. Pathway analysis of these proteins identified factors involved in the vesicle-mediated delivery of integrin signalling proteins. These findings indicate that BM-MSC EVs provide an effective support system for ASC survival and IgG secretion.

## Introduction

The interrelationship between bone marrow (BM) mesenchymal stromal cells (MSC) and antibody secreting cells (ASC), or plasma cells (PC), has been explored in a variety of *in vitro* models, suggesting that MSC impart various growth factors, cytokines, and chemokines to maintain survival and function of B-lineage cells [1–6]; however, these studies have relied upon the use of cancer-derived, transformed B-lineage cells. The survival mechanisms of the BM microniche are thought to be mediated by local paracrine MSC secretion of IL-6 and vascular endothelial growth factor (VEGF) [1–3] as well as adhesion or cell-cell contact [4]. Others have shown that some of these effects can occur via MSC-derived extracellular vesicles (EVs), such as exosomes and microvesicles (MVs) [5]. However, the ability of MSC-derived EVs to support the *ex vivo* function of non-transformed peripheral blood (PBL)-derived ASC has not been completely described.

MSC are a low-frequency population in the adult marrow, comprising of approximately 1 in 10,000 mononuclear cells isolated from an BM aspirate [6–9]. The long-lived PC also take up residence in the BM, and they too are quite rare (accounting for only about 0.05% of all marrow mononuclear cells) [10]. Thus, communication between these two such rare BM populations is likely to require cell-cell contact, chemokine gradients, close paracrine signalling or other cell-contact-independent mechanisms. With the advent of MSC-based cell immunotherapies, it has been postulated that MSC trapped in the lungs rely in part on the release of EVs to deliver both soluble and membranous proteins over distances in order to mediate therapeutic efficacy [11,12]. Whether these mechanisms also apply to normal communication between the healthy BM-MSC and ASC in the vast BM compartment required further exploration.

BM-MSC have been shown to provide survival factors in the human BM microniche that support long-lived PC survival [13–16]. Co-culture systems of marrow stromal cells with PC showed that IL-6 and fibronectin (FN-1) were two soluble factors needed for effective long-term immunoglobulin secretion [14]. Additionally, secreted factors such as IL-6 alone were necessary but not sufficient for antibody production [17]. Subsequent reports have shown contact-dependent signalling, via molecules like CXCL12 and the integrin α_4_β_1_ (VLA-4), to impart important cues delivered by MSC, suggesting that cell-cell contact or close proximity may be required in the BM microniche in addition to secreted factors [18–20]. However, our group recently developed a novel *in vitro* PC survival system that models the BM microniche. It reveals the critical role of the BM-MSC in maintaining survival of *ex vivo* ASC for over 50 days in culture (Nguyen et al., submitted). Most interestingly, cell-cell contact was not required as the MSC secretome, or supernatant, was sufficient to maintain ASC functionality. In pursuit of a reductionist cell-free platform, we sought to address if supernatant-derived EVs alone could recapitulate this phenomenon.

EVs are small membranous spheroids that can be released from a variety of cell types. They feature distinctive tetraspanins at their membrane surface (such as CD9, CD63, and CD81) and transport cargo, including proteins and RNA, over short or long distances. EVs can be secreted from cells as large MVs (100–1000 nm diameter) or as the nanoscale exosomes (30–150 nm diameter) [21]. Larger-sized MVs are released from cells as outpouchings of plasma membrane, whereas exosomes have trafficked through the cell’s multivesicular body, part of the endosomal sorting complex required for transport (ESCRT), which tags, sorts and matures endosomes with the use of membrane-bound Rab GTPases [22]. A variety of reports have explored MSC-derived EVs as an avenue for cell-free cell-based therapy, showing therapeutic efficacy in animal models of liver and heart disease [23,24]. In this study, we demonstrate that MSC-derived EVs indeed provide a cell-free component to recapitulate the marrow niche and a novel mechanism of communication between hematopoietic stroma and ASC, thereby enabling the *ex vivo* cultures of healthy human PC.

## Results

### Secretomes from non-irradiated and irradiated BM-MSC promote ASC survival *ex vivo*

Healthy adults were enrolled for BM aspirates from five subjects (ages 19–55 years old, 3 males, 2 females). BM-MSC were cultured and secretomes were prepared from irradiated and non-irradiated BM-MSC (Supplemental Figure 1) to compare the effects of irradiation on the contents of secretomes for survival of human ASC. We had begun initial studies using growth-arrested MSC (irradiated), as the technique is typically used to prevent these cells from overgrowth of long-term co-cultures with PC. However, as the present study did not involve direct co-culture of the two cell types (but rather the secretome), we decided to also characterize the effects of secretomes from MSC in their proliferative phase (non-irradiated). Although some reports have shown irradiated MSC maintain their immunomodulatory function *in vitro* [25,26], we still hypothesized that radiation injury, or entry into a non-replicative G_0_ cell cycle, may have detrimental effects on the ASC-support engendered by our secretome; thus we sought to test secretomes from both irradiated and non-irradiated MSC.

**Figure 1.**
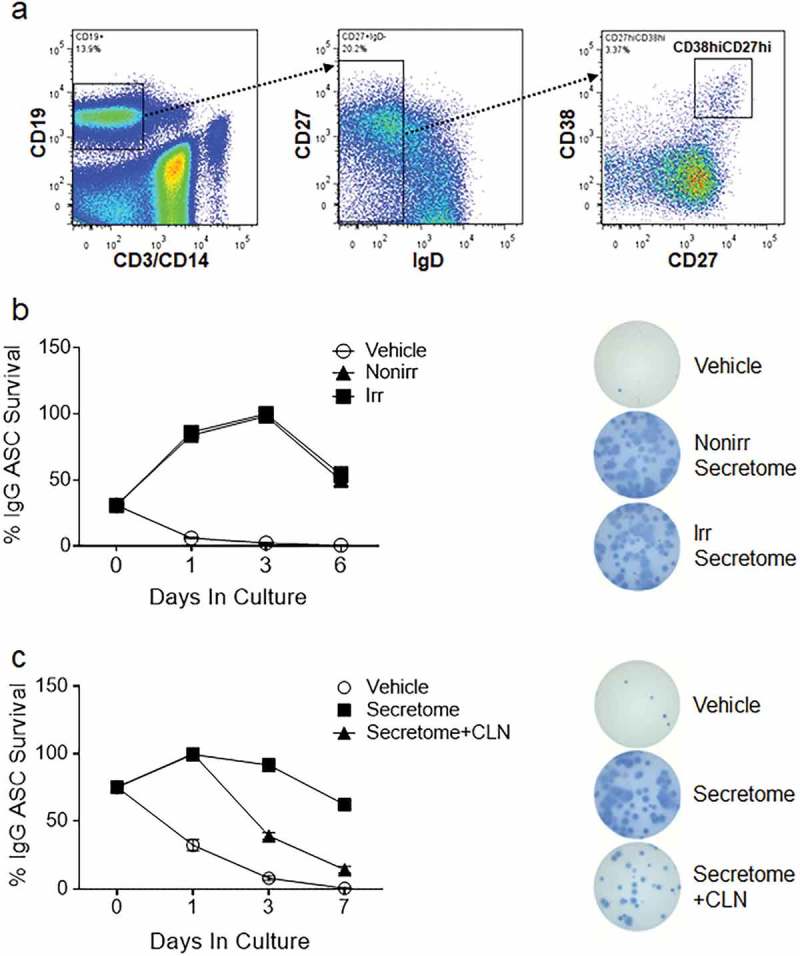
ASC survival *ex vivo* with irradiated, non-irradiated secretomes and secretomes treated with Cleanascite. (a) Representative flow cytometry analysis of blood ASC (CD19^+^CD27^hi^CD38^hi^) from PMBC isolated 7 days after vaccination from a healthy adult. (b) Blood ASC from a healthy adult after Tdap-vaccination cultured in vehicle (R10) and secretomes from non-irradiated and irradiated BM-MSC. Each culture well was seeded with ~1,913 (for vehicle) or ~1,275 (for secretomes) ASC. Cells were harvested at days 0, 1, 3 and 6 post-culture and the frequency of IgG-secreting ASC were measured by Elispot assays; the spot numbers were normalized to maximal ASC (100%) as determined by each individual experiment. Shown on the right are representative Elispot images (at day 3). (c) Blood ASC from a healthy adult at the peak of ASC frequencies (7 days after rabies immunization) were cultured in vehicle (R10), secretome from irradiated BM-MSC, and secretome from irradiated BM-MSC that had been pretreated with Cleanascite. Each culture well was seeded with ~1,500 ASC. Cells were harvested at days 0, 1, 3, and 7 post-culture and the frequency of IgG-secreting ASC were measured by Elispot assays; the spot numbers were normalized to maximal Elispot frequencies (100%) as determined by each individual experiment. Representative Elispot images (at day 3) are shown. Experiments in Figure 1(b,c) are representative of > 3 experiments. Nonirr, non-irradiated; Irr, irradiated; CLN, Cleanascite.

It is well established that ASC peak in the blood after vaccination and so we recruited healthy adults after vaccination. For secondary immunization, ASC can be found in circulation approximately 7 days after the vaccine [27]. To measure the secretome’s activity, ASC (CD19^+^CD27^hi^CD38^hi^) were FAC-sorted (Figure 1(a)) from the PBL of an additional healthy adult 7 days after Tdap vaccination and cultured in conventional media [RPMI + 10% foetal bovine serum (FBS) (R10, or vehicle)] or the secretomes from irradiated and non-irradiated MSC cultures. ASC survival in the secretomes was measured by IgG ASC Elispots as a percentage of the peak frequency, usually seen during days 1–3. There was similarity in IgG ASC survival between the secretomes of non-irradiated and irradiated MSC (84–86%, 98–100% and 50–54% on days 1, 3 and 6, respectively) (Figure 1(b)). Of note, the increase in ASC survival on day 1 was due to revival of ASC IgG secretory function after stress of FAC sorting rather than increased proliferation as shown by negative BrdU labelling experiments (Nguyen et al., submitted). Despite irradiation, the capacity of the MSC secretome remained intact to support ASC survival.

### Lipid disruption of BM-MSC secretome abrogates support to ASC survival

EVs are small membrane vesicles secreted by MSC through fusion of multivesicular bodies with a lipid plasma membrane. To understand whether disrupting the lipid plasma membrane that upsets the integrity of the EVs would compromise the survival activity of either the non-irradiated or irradiated secretomes, we again sorted ASC from the PBL of another healthy adult 7 days after rabies vaccination. We then cultured ASC with conventional media (vehicle), secretome from irradiated MSC, or secretome from irradiated MSC that had been pretreated with the lipid-disrupting agent Cleanascite [28], which is known not to alter protein functionality [29]. Cells were harvested at days 0, 1, 3 and 7 post-culture and the frequency of IgG ASC Elispots were measured. Compared to the untreated irradiated MSC secretome, there was a rapid decline in ASC survival when cultured in the vehicle alone, falling below 10% by day 3 (Figure 1(c)). Cleanascite-treatment of the secretome dramatically reduced ASC functional survival, from 92% to 39% by day 3 and from 62% to 14% by day 7 (*p* = 0.02, two-way ANOVA) (Figure 1(c)). Similar reductions were also noted with the secretome of non-irradiated MSC when treated with Cleanascite (at day 3; data not shown). These results demonstrate that lipid-membrane bodies, such as EVs, could mediate important ASC survival factors within the MSC secretome.

### Quantification of EVs

**Figure 2.**
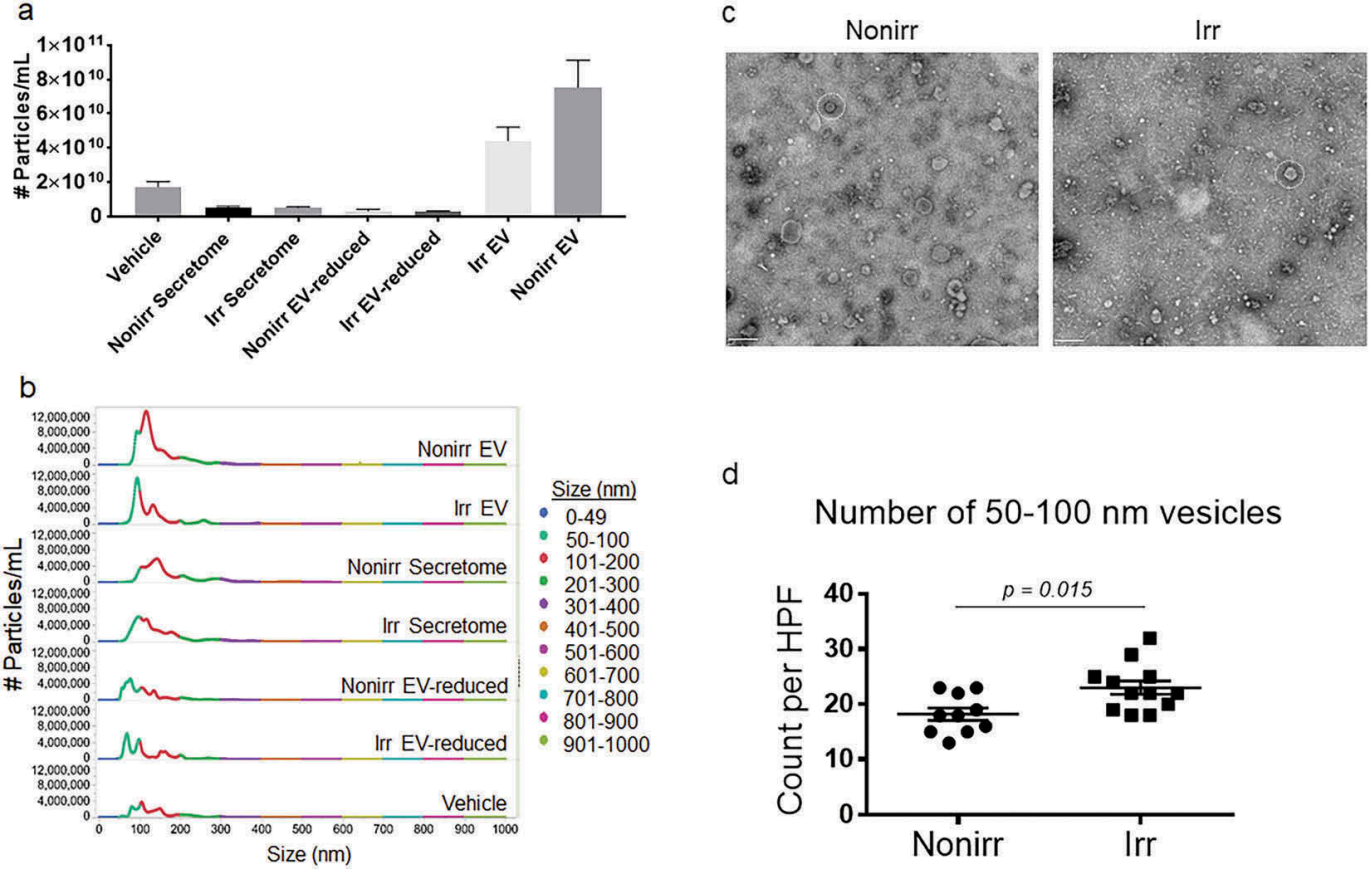
Quantification and EM of EVs isolated from irradiated and non-irradiated secretomes. (a) Quantification of particles from EV fractions and EV-reduced fractions of non-irradiated and irradiated MSC secretomes by nanosight. (b) Distribution of the concentration of various size particles from non-irradiated and irradiated EV fractions and EV-reduced fractions, whole secretome, and vehicle controls. (c) Representative whole-field EM images for EV fractions isolated from irradiated or non-irradiated BM-MSC secretomes. Imaging was performed using uranyl-oxalate negative staining and observed using a JEOL Transmission Electron Microscope. Twelve grids were prepared and imaged and counted by observers who were blinded to the treatment status. Scale bar indicates 200 nm. EVs are 50–100 nm in size, and putative vesicles are indicated by white dashed lines. (d) Ten to twelve high-power fields were enumerated for 50–100 nm vesicles by blinded observers (*p* = 0.015; Mann–Whitney test). Nonirr, non-irradiated; Irr, irradiated.

EV concentrations from irradiated and non-irradiated MSC secretomes were quantified using nanosight tracking analysis (NTA), and concentrations were 5.1 × 10^9^ and 5.2 × 10^9^ particles/mL, respectively (Figure 2(a)). To ensure EVs originated from MSC and not FBS, we used EV-depleted media to prepare MSC secretomes prior to EV isolation. Concentrations of EVs in the irradiated and non-irradiated EV fractions were almost 10-fold higher at 7.5 × 10^10^ and 3.5 × 10^10^ particles/mL, whereas the EV-reduced fractions were significantly depleted (3.1 × 10^9^ and 2.9 × 10^9^ particles/mL in the non-irradiated and irradiated EV-reduced fractions, respectively). Although the concentration of particles were higher in the non-irradiated EV fractions, the percentage of 50–100 nm particles were lower in non-irradiated (15%) vs irradiated fractions (40%), suggesting that there may be increased concentrations of EVs in the irradiated EV fractions (Figure 2(b)). Additionally, the concentration of particles 101–200 nm in size within the non-irradiated EV fractions (70%) was higher compared to the irradiated EV fractions (50%) suggesting more MVs in the non-irradiated fractions. Quality of the EVs differed with potentially higher EV concentrations in the irradiated EV fractions compared to non-irradiated fractions. The vehicle (R10) with FBS which was not depleted of EVs had slightly higher concentrations of EVs compared to both the irradiated and non-irradiated total secretomes which were generated from EV-depleted FBS, albeit all were quite low. Of note, the vehicle (R10) had a small amount of EV contamination from untreated FBS but it conferred no biological activity (Figure 1). In summary, the nanosight measurements confirmed that the EV fractions indeed had higher concentrations of EVs compared to EV-reduced fractions, and the quality of the EVs differed significanty. Finally, despite small amounts of EV contamination from untreated FBS in the vehicle control, it offered no ASC survival activity.

### Electron microscopy (EM) shows vesicular size consistent with EVs

We used EM to verify the size and morphology of EVs in the EV fractions [30]. Figure 2(c) shows representative whole-field images, which demonstrate that the size of these vesicles was consistent with EVs. However, EV frequencies of 50–100 nm vesicular bodies were higher in the irradiated versus non-irradiated EV fractions (*p* = 0.0159; Mann–Whitney test) (Figure 2(d)), which was consistent with the nanosight results (Figure 2(a,b)).

### Immunogold EM confirms EV markers

**Figure 3.**
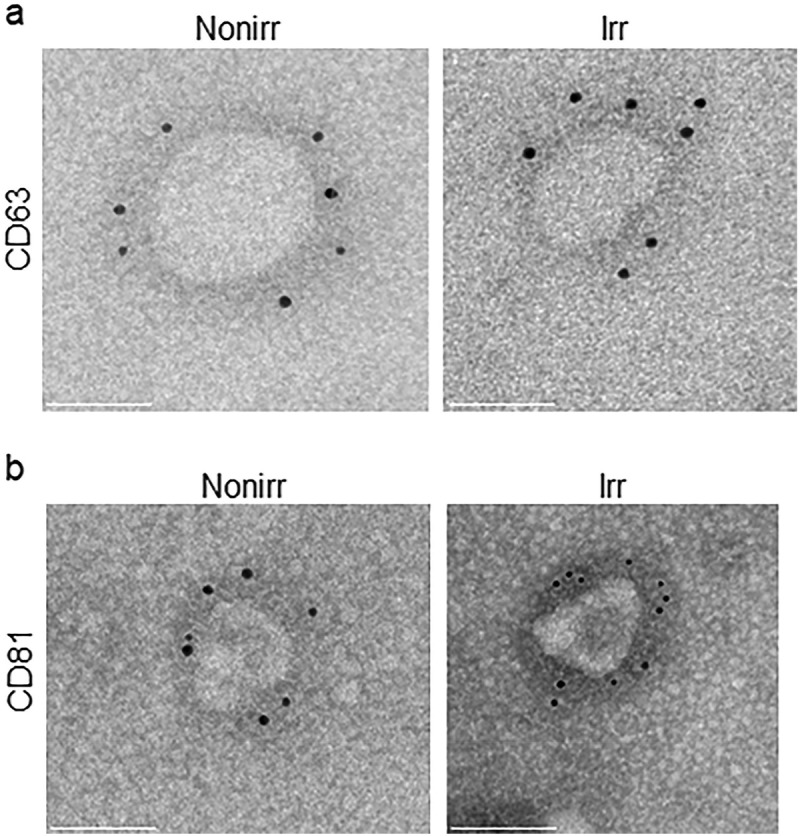
Immuno-gold EM of EV markers. Immuno-gold-labelling EM confirms 50–100 nm vesicles, referred to as EVs. Immuno-gold labelling with (a) CD63 and (b) CD81 on ultrastructure of EVs appeared comparable for those derived from non-irradiated and irradiated BM-MSC. Scale bar indicates 50 nm. These micrographs are representative of over two dozen such images using EV fractions derived from three donors. Nonirr, non-irradiated; Irr, irradiated.

As NTA and EM only assessed vesicles based on size and not protein or cargo identity, we sought a more specific method for characterization. Immuno-gold EM is an important tool to assess the presence of known EV markers. Thus, we performed immune-gold EM to assess if the vesicles expressed CD63 and CD81, two markers known to be found on MSC-derived EVs [31,32]. Representative images are shown in Figure 3(a) (CD63) and (3b) (CD81), which confirm EV markers on these vesicles. The ultrastructure of the vesicles was similar whether they were derived from irradiated or non-irradiated MSC.

### Endosomal markers TSG-101 and ALIX were negative

Although there were high concentrations of 50–100 nm vesicular bodies in the EV fractions, the endosomal markers ALIX and TSG-101 were negative by Western blot analysis (data not shown) for both irradiated and non-irradiated fractions, suggesting that EVs between 50–100 nm may not all comprise of exosomes. Lack of sensitivity of the Western blot analysis may have been an issue since HeLa whole cell extracts were positive at 100 and 20 ng and barely detectable at a low protein concentration (4 ng), which was the maximal concentration of the EV preparations. Although some particles of 50–100 nm may have in fact been exosomes, they could not be confirmed by Western blot analysis. Thus, herein we refer to them as EV fractions (Supplemental Figure 1).

### EVs support ASC function irrespective of cell growth status

**Figure 4.**
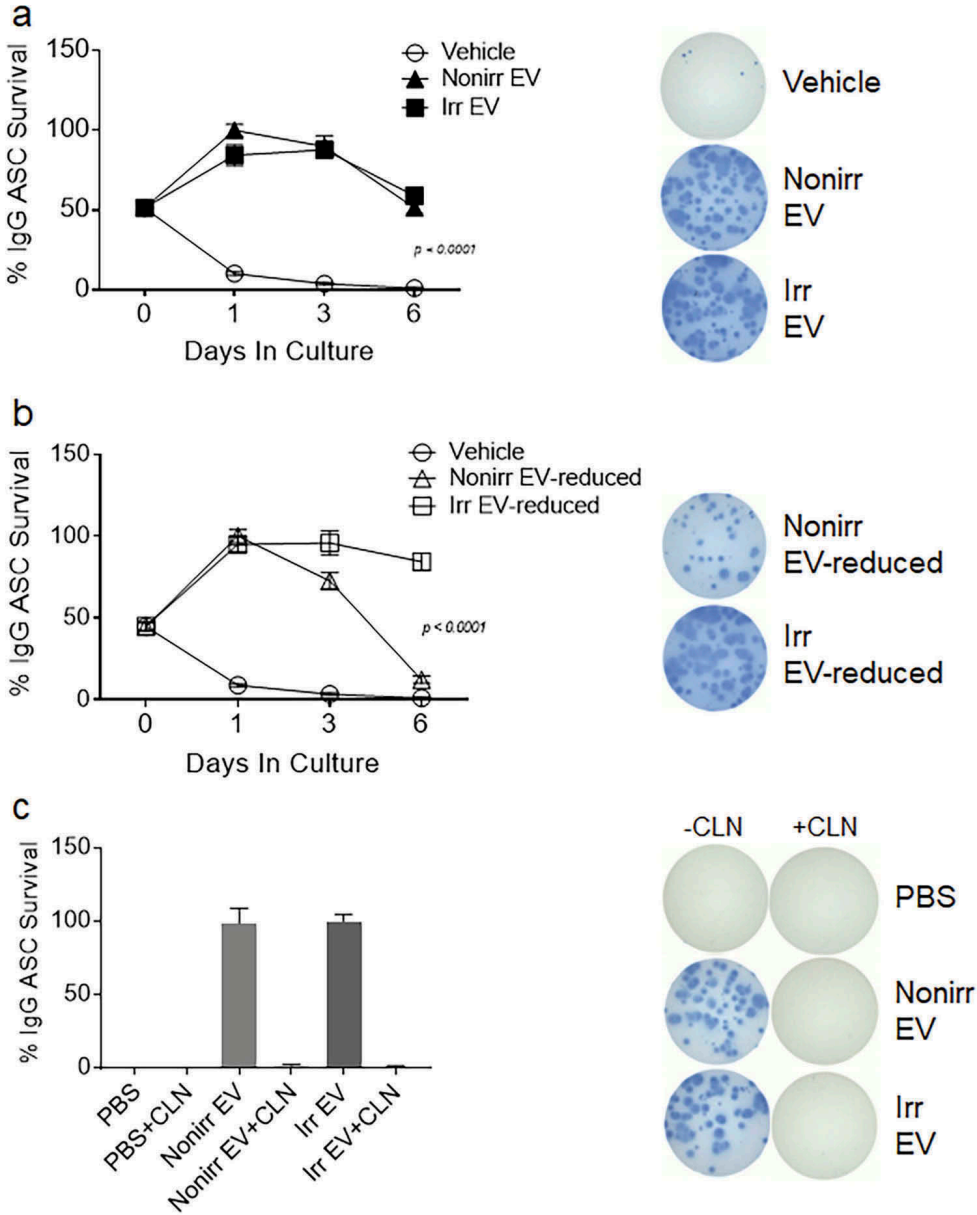
Both irradiated and non-irradiated BM-MSC-derived EVs support ASC survival. (a) Blood ASC were isolated from a healthy adult 7 days after Tdap vaccination and cultured in vehicle (R10) or EV fractions from irradiated or non-irradiated BM-MSC secretomes. Each culture well was seeded with ~1,913 ASC. Cells were harvested at days 0, 1, 3 and 6 post-culture and IgG Elispots were performed. IgG-secreting ASC spot frequencies were normalized to maximal ASC Elispots (100%) (*p* < 0.0001, two-way ANOVA). Shown on the right are representative photomicrographs (at day 3). The figure shows the representative data from three independent experiments. (b) IgG spot frequencies from ASC cultured in vehicle (R10) or EV-reduced fractions from irradiated and non-irradiated BM-MSC secretomes. Each culture well was seeded with ~1,913 (for vehicle) or ~1,275 (for EV-reduced fractions) ASC. Cells were harvested at days 0, 1, 3 and 6 post-culture and IgG Elispots were performed. IgG-secreting ASC spot frequencies were normalized to maximal ASC Elispots (100%) (*p* < 0.0001, two-way ANOVA). Shown on the right are representative photomicrographs (at day 3). (c) Blood ASC from two healthy adults were cultured in PBS and EV fractions from irradiated or non-irradiated BM-MSC secretomes, with and without pretreatment with Cleanascite. Each culture well was seeded with ~829 or ~927 ASC. Cells were harvested at days 0, 1 or 3 post-culture and the frequency of IgG-secreting ASC were measured by Elispot assays; the spot numbers were normalized to maximal Elispot frequencies (100%). Representative Elispot images (at day 3) are shown. Nonirr, non-irradiated; Irr, irradiated; CLN, Cleanascite.

EVs derived from either irradiated or non-irradiated MSC were co-cultured with ASC, and IgG Elispot assays were performed on day 1, 3 and 6 (Figure 4(a)). Again, we observed a rapid drop in ASC survival when the cells were cultured in conventional media (vehicle), with only 10% of cells still secreting at day 1 and 4% by day 3. In contrast, the EV fractions derived from non-irradiated MSC afforded Elispot frequencies of 100%, 90% and 51% on days 1, 3 and 6, respectively. In a similar fashion, the EV fractions from irradiated MSC were also capable of supporting the ASC at 84%, 88% and 59%, at days 1, 3 and 6, respectively. To demonstrate the role of EVs in the non-irradiated secretomes, we show that EV-reduced fractions showed decreased ASC survival. These data suggest that EVs support ASC function irrespective of cell growth status.

Interestingly, in the irradiated secretomes, both secretory factors and EVs appear to play a role in ASC survival. For the irradiated secretome, no difference between the EV fractions and EV-reduced fractions was found in ASC survival activity, suggesting that other factors may also be redundant (Figure 4(a,b)). Nanosight quantification showed that the EV-reduced fractions were 10-fold lower in EV concentrations. Thus, we concluded that soluble secretory factors (such as lipoproteins) from irradiated MSC in the EV-reduced fractions probably play redundant roles for ASC survival. To summarize, we found that the EV fractions from either non-irradiated or irradiated cell sources were equally competent to support ASC function when compared to the vehicle alone (*p* < 0.0001; two-way ANOVA). However, for irradiated MSC secretomes, soluble factors also play an important role in ASC survival.

To definitively show that EVs and not protein factors in the EV fractions (irradiated and nonirradiaed) provided ASC survival, we disrupted the EV fractions by pretreating with Cleanascite before culturing with ASC. We found that by day 3, the Cleanascite-treated EV fractions had abolished any ASC survival activity (Figure 4(c)). This was consistent for both the irradiated and non-irradiated EV fractions. To also ensure that Cleanascite did not offer any survival advantage, PBS pretreated with Cleanscite was cultured with ASC, and no Elispots were detected. These data suggest that lipid disruption within the EV fractions abolished biologic acitivity, demonstrating that EVs and not proteins provided ASC survival.

### Proteomics and ingenuity pathway analysis (IPA)

**Figure 5.**
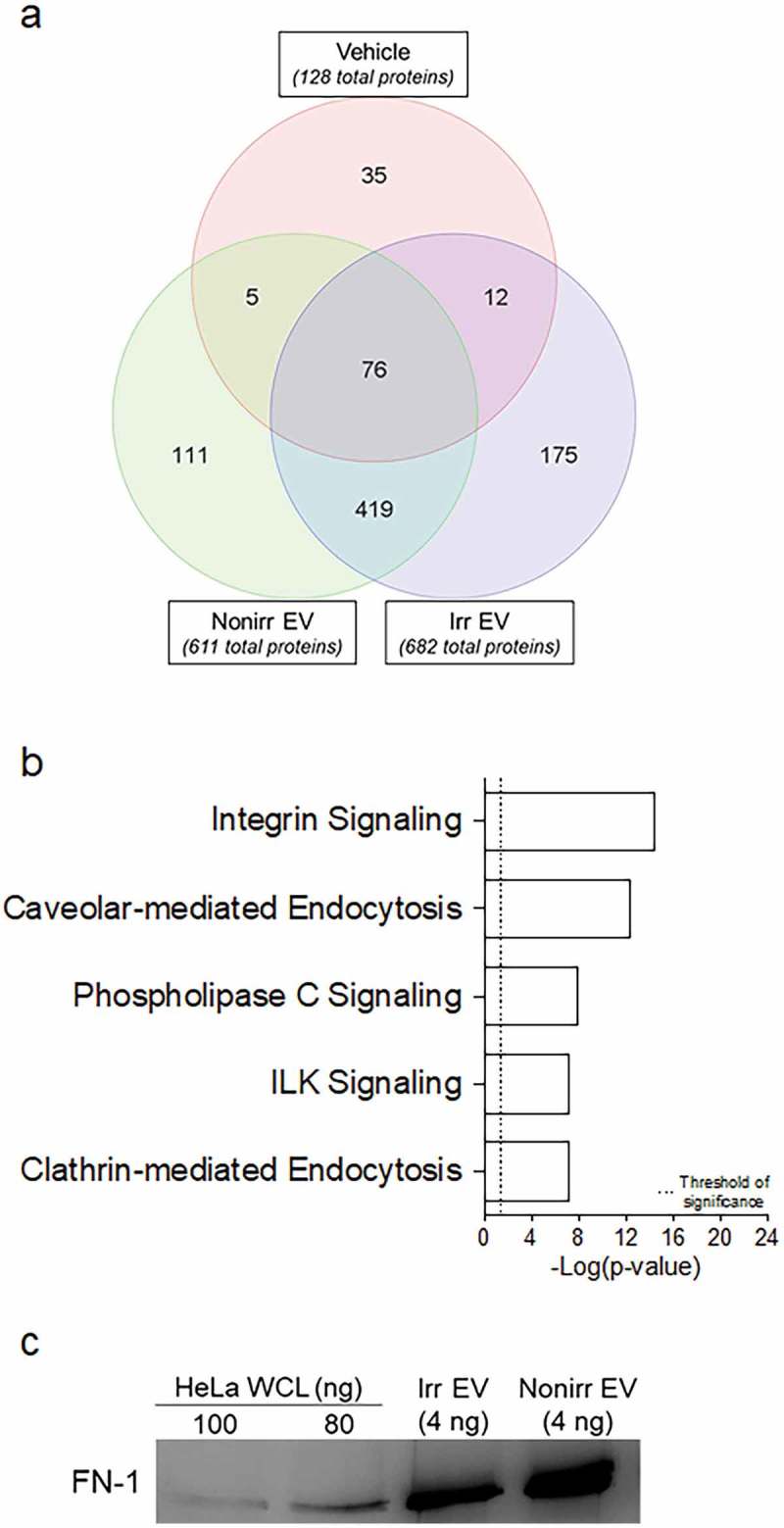
Proteomics of irradiated vs. non-irradiated EVs. (a) The 419 common proteins found in both EV fraction types were used as input for IPA. (b) Curated list of five of the most significantly represented pathway lists generated by IPA. Within the IPA platform, a pathway was considered statistically significant according to the proportion of pathway members present. A statistically significant *p*-value of 0.05 is equivalent to a – log(*p* = 1.35), which is indicated on the plot as a vertical dashed line, at the x-value of 1.35. (c) Western blot analysis of fibronectin-1 (FN-1) from both irradiated and non-irradiated EV fractions. HeLa whole-cell lysates (WCL) were used as positive controls. Nonirr, non-irradiated; Irr, irradiated.

To identify the protein factors in the EV fractions and hypothesize how they may mediate ASC survival, we performed proteomic analysis on the EV fractions derived from sample-matched pairs of actively proliferating (non-irradiated) or growth-arrested (irradiated) BM-MSC. As controls, we used vehicle (R10; conventional media). Proteins were extracted and digested and analyzed via mass spectrometry-based proteomics (as described in Methods; complete protein lists are included in Supplemental Table 1). Six hundred eighty-two (682) proteins were identified in the irradiated EV fractions and 611 in the non-irradiated EV fractions, compared to 128 in the vehicle. Proteins that were uniquely common to both the irradiated and non-irradiated EV fractions, but not found in the vehicle, were used as input for further analysis using IPA (Figure 5(a)). A curated list featuring five of the most highly significant pathways associated with the EV proteomes is presented in Figure 5(b) (full list may be found in Supplemental Table 2). We note significant representation for both caveolar- and clathrin-mediated endocytosis, identified by proteins including clathrin light chain A (CLTA), the coatomer protein complex (COPA), and a number of integrins including the integrin β1 subunit. IPA also revealed statistically significant representation for integrin and integrin-linked kinase signalling, with one important protein identified as cell division control protein 42 (Cdc42). The last pathway we noted to be of significant interest was phospholipase C (PLC) signalling, identified by the presence of proteins including the Ras-like proto-oncogenes A (RALA) and B (RALB).

To validate the proteins, we performed Western blot analysis with FN-1 and Cdc42 and confirmed the presence of immunoreactive FN-1 in both irradiated and non-irradiated EV fractions but could not detect Cdc42 (Figure 5(c)). Although the proteomics is not quantitative, we had greater number of peptide “hits” with FN-1 versus Cdc42 (see Supplemental Table 1), thus additional validation may require more sensitive techniques (such as ELISA), as demonstrated by Sze et al. [33]. Taken together, the identification of these proteins and their corresponding pathways suggest that BM-MSC-derived EVs may be capable of delivering the relevant immune-activing signals to receiver cells such as ASC.

## Discussion

In this study, we demonstrate that EVs isolated from BM-MSC secretome sustain, support and enhance function of human IgG ASC. We found that irradiation, which had been initially used to growth-arrest the MSC, avoiding overgrowth by these robustly proliferative cells, did not alter functional survival-conferring capacity nor the yield of MSC-derived EVs. We hypothesized that irradiation would modify the secretome of BM-MSC relative to replication-competent cells, and that the EVs derived from these growth-arrested cells might provide differential *in vitro* support to ASC. Indeed, published reports have shown that irradiation of a cell type can quantitatively alter protein or RNA content of EVs, with resultant differences in downstream biological applications [34–36]. However, we report that EVs from either cell source (irradiation or non-irradiation) can be used with equivalent efficacy to support ASC *in vitro*.

Quantification of the particles showed increased EVs in both non-irradiated and irradiated EV fractions although our preparations had higher concentrations of particles from non-irradiated MSC. Interestingly, the non-irradiated fractions had higher concentration of larger particles compared to the irradiated EV fractions. Additional immune-gold EM staining with CD63 and CD81 was important to assess the presence of known EV markers. Positive CD63 and CD81 staining of particles 50–100 nm in size suggested that some may be exosomes, despite negative ALIX and TSG-101 on Western blot analysis, which is known to have low sensitivity. In summary, by size and immune-gold EM, we demonstrate that EV fractions from both non-irradiated and irradiated MSC support ASC survival.

The therapeutic dosing of EVs in the cell therapy in the literature can be challenging to interpret, as quantities are often calculated via traditional spectrophotometry rather than ELISA. One example is a 2013 report from Li et al., in which the team used a BCA protein assay to quantify EVs, noting a therapeutic effect on murine liver inflammation after administration of EVs at a concentration of 750 ng/µL (but no total dose is provided) [37]. Also in 2013, Tomasoni and colleagues reported therapeutic effect on renal tissues after dosing mice with EVs at a concentration of 25 ng/µL, again with no full dose reported [38]. Whereas Li et al. used a BCA protein assay, Tomasoni et al. used a Bradford protein assay, both of which should be considered as upper bounds of an EV dose, as ultracentrifugation may carry-over non-EV proteins.

In our study, for non-irradiated secretomes, EV concentrations appeared to play a major role in ASC survival since the EV fractions supported ASC survival while the EV-reduced fractions were clearly inferior and only slightly better than media alone (particularly during the late culture periods). However, the results for the irradiated secretomes were more complex since both the EV fractions and the EV-reduced fractions supported ASC survival, suggesting two possible interpretations. First, both EVs and secretory proteins are important for ASC survival and the quality of the proteins differ in the non-irradiated and irradiated secretomes. Irradiated MSC, in fact, may secrete critical survival factors that are unique and carry-over in EV-reduced fractions compared to non-irradiated EV-reduced fractions. A second interpretation could be that EVs are not critical for ASC and only soluble carry-over proteins are essential for ASC survival. If only carry-over proteins were involved, it would be difficult to understand the loss of ASC survival with Cleanascite-treatment in both the irradiated and non-irradiated EV fractions. In conclusion, both non-irradiated and irradiated EV fractions support ASC; however, irradiated EV-reduced fractions also retain unique secretory survival proteins that can play redundant roles in ASC survival.

As MSC and ASC are both relatively rare populations, we hypothesized that the release of EVs might serve as an intermediary for short- or long-distance signalling. Indeed, our laboratory has shown that MSC can take up residence in lung tissue, whereupon clinical or biologic activity at distant sites can still be observed [39,40]. The observations that cells may indeed communicate between distant organ sites via EVs led us to hypothesize that these vesicles may also be a novel model for ASC-MSC interactions within the vastness of the marrow niche.

Our proteomic approach was hypothesis-generating in regards to the MSC factors that support ASC *in vitro*; future analyses may be focused on the mRNA and microRNA carried within the MSC-derived EVs. In analyzing the proteomic and pathway datasets, we sought to model MSC-to-ASC crosstalk, but with the acknowledgment that such is a one-directional analysis. Agnostic to the transcriptional and translational events occurring within the ASC compartment, our model is best suited to explore how MSC-derived EVs may recapitulate the stromal support of the marrow niche. Of note, known survival factors such as IL-6 and VEGF were not found as others also showed [33]. It is also quite possible that our methods may not have had the sensitivity needed for identifying low-abundance proteins, as others have suggested that such small molecules are not readily detectable by mass spectrometry [41]. Nonetheless, over 400 candidate proteins overlapped in both irradiated and non-irradiated EVs. Within the proteomes, we identified the presence of pathways consistent with vesicular transport and targeted uptake by recipient cells, including the clathrin-mediated and caveolin-mediated signalling pathways. Both of these pathways have been shown to play a role in the uptake of EVs by B cells and PC, consistent with EV-mediated delivery of survival factors derived from stromal cells [42,43].

The EVs’ proteomic prevalence of integrin and the integrin-linked kinases is an important finding, as these molecules have already been shown to be key contact-dependent mechanisms whereby stromal cells support lymphocyte functionality. For instance, the integrin α_4_β_1_ (VLA-4) has been described as a key factor for the generation of long-lived PC [14,44]. Reports show that these cells rely on a combination of soluble and contact-mediated mechanisms to fulfill hematopoietic and immunomodulatory functions [45–47]. EVs may help explain these phenomena by parsimony; if coated with bioactive integrins, they may be capable of triggering membrane-associated signalling cascades while also delivering soluble protein cargo to target cells. Future studies can elucidate if the integrins themselves promote ASC survival or merely deliver packaged survival factors.

Plasma membranes, like the surfaces of EVs, are composed of a phospholipid bilayer, decorated with a variety of bioactive enzymes, lipids and sugars, that enable cellular recognition. Immunological cell signalling cascades often begin at the membrane surface, where phospholipid substrates like PIP_2_ (phosphatidylinositol 4,5-bisphosphate) are cleaved by a class of molecules known as the PLC family, generating downstream signalling molecules that activate cellular transcription programs for proliferation and differentiation in a variety of cell types, including lymphocytes [48]. In our proteomic assessment of EVs, we noted the significance of the PLC signalling pathway, including the presence of the signalling proteins RALA and RALB. These proteins are both guanosine triphosphatases (GTPases) and act in close association with G-protein coupled receptors to transduce signalling events via GTP hydrolysis. RALA and RALB are both important for the proliferation of immune cells as well as membrane trafficking and exocytosis; of particular note, RALA is required to suppress apoptosis [49]. An additional GTPase identified in EVs was Cdc42, which activates actin polymerization in target cells, coordinating cell migration, proliferation, and survival [50]. Recent reports have shown that Cdc42 to be essential for the activation and function of mature B cells in a mouse model of primary immune deficiency [51].

In conclusion, this study shows that EVs derived from the BM-MSC secretome enhance *in vitro* human ASC survival. The mechanisms of the MSC-ASC communication may occur in close proximity via local paracrine interactions and over fairly long distances via EVs. Our proteomic analysis suggests that MSC EV protein cargo contains a number of known molecules related to immune cell proliferation, protein translation, endocytosis, and integrin signals. The pathways identified herein offer possible candidates for short interfering RNA knockdown or antibody-neutralization. Such steps will help narrow the search for key factors that maintain survival of human PC in the long-lived PC niches.

## Materials & methods

### Human subjects

We recruited a total of 17 healthy adults (8 females and 9 males) for either PBL samples (*n* = 12) or BM aspirates (*n* = 5) with a mean age of 35 ± 12 years of age. All studies were approved by the Emory University Institutional Review Board Committee. The 11 PBL samples were obtained from healthy adult subjects (mean age of 37 ± 11 years old), including who received one or more routine vaccines, which included rabies and Tdap at 7 days after immunization. Sample collection dates ranged from 2014–2018.

### MSC isolation and culture

Human MSC were isolated from BM aspirates collected from the iliac crest of consenting volunteer subjects [52]. BM aspirates were diluted 1:2 with PBS and layered onto a Ficoll density gradient to isolate mononuclear cells. The cells were centrifuged at 400–500 × *g* for 10–20 min and thereafter plated in complete human MSC medium (α-MEM, plus 10% FBS or FCS), or 10% human platelet lysate (hPL), plus 100 U/mL penicillin/streptomycin (Corning) at 200,000 cells/cm^2^. Non-adherent hematopoietic cells were removed by changing the medium after 3 days of culture and MSC were allowed to expand for 7 days at 37°C in 5% CO2. Thereafter, the cells were passaged weekly and reseeded at 1,000 cells/cm^2^. All experiments were performed with MSC at passage 3 or 4. Although some cultures were first expanded in (α-MEM+hPL), all subsequent cultures were performed in M10 (see below).

### Culture medium preparation

MSC were cultured in a special culture medium in which serum-derived EVs were reduced. It is important to hereby qualify that this prepraration was designed to deplete EVs from culture media in order to develop appropriate negative and positive controls. However, overnight ultracentrifugations may only reduce, not 100% deplete, contaminating EVs [53]. While this assertion is important to qualify, such ultracentrifugation methods were pursued in order to best-develop matched controls and experimental designs. The initial culture medium was M20 (α-MEM plus 20% FCS plus 100 U/mL penicillin/streptomycin (Corning)). All centrifugations of all liquids in this project occurred with new tubes and caps that had been rinsed twice with 70% ethanol, twice with PBS, and then left to air dry overnight in a biosafety cabinet under constant ultraviolet light exposure. M20 medium was transferred to polyallomer tubes and then spun at 100,000 × G in a Beckman Optima L-80XP Ultra Centrifuge at 4°C for 16–18 h. The pellet containing serum-derived EVs was discarded and the supernatant was extracted using a sterile syringe fitted with a 21 G needle. The supernatant was then mixed 1:1 with sterile serum-free media to prepare a final mixture of 10% α-MEM (M10). This resulting serum-derived EV-reduced M10 was then passed through a 0.2 µm bottle-top vacuum filter system (Corning) and stored at 4°C until use. All centrifugations occurred at 4°C unless stated otherwise.

### Purification of EVs

After initial tissue culture expansion as described, MSC were washed with PBS, trypsinized, and resuspended in M10, with some preparations then exposed to a total of 30 Gy irradiation. Cells were then replated into new 150 or 175 cm^2^ tissue culture flasks, at a density of 8.5 × 10^6^ cells per flask, and placed into separate tissue incubators. Every 24 h, the conditioned medium (secretome) from each flask was aspirated and replaced with fresh M10. Secretome from each irradiation treatment group collected all week was pooled into sterile bottles and stored at 4°C. The pooled secretome was transferred into sterile 50 mL polypropylene tubes (Corning) and spun at 300–500 × G for 10–15 min; the supernatant was collected and then spun again in 50 mL tubes at 2,000–10,000 × G for 20–30 min. The resulting supernatant was then transferred to freshly cleaned polyallomer tubes and spun at 10,000–20,000 × G for 60 min in a Sorvall RC-6 Plus Centrifuge. The pellets were discarded, and the supernatant was transferred to freshly cleaned polyallomer tubes and then spun 100,000 × G for 70 min in the Beckman Optima L-80XP Ultra Centrifuge or Beckman Coulter Optima XL-100K Ultracentrifuge. We refer to the supernatant resulting from this spin as the EV-reduced fractions (Supplemental Figure 1), and 500–1,000 µL aliquots were taken and stored at −80°C until use. The pellets from all condition-identical tubes were then washed with sterile PBS, pooled together and spun again at 100,000 × G for 1–3 h in the Beckman Optima L-80XP Ultra Centrifuge or Beckman Coulter Optima XL-100K Ultracentrifuge. The supernatant was aspirated using a sterilized glass Pasteur pipette, with the resulting pellet, which we refer to as EV fractions (Supplemental Figure 1), resuspended in 500–600 µL sterile PBS and stored at −80°C until use. Our method, which did not use a sucrose gradient, was adapted from other similar publications [30,54].

### NTA

The size distribution and concentration of EVs was determined in each fraction using a Nanosight NS300 instrument from (Malvern). Prior to data acquisition, each fraction was diluted 1:10 or 1:100 with sterile PBS to ensure that the number of detectable particles was within the optimal range of 10^7^–10^9^ particles/mL. After diluting, each fraction was placed into 1 mL syringe before placing on the instrument. Five 60 s frames were acquired for each sample to ensure accurate quantitation of the sizes and concentrations of EVs in each fraction using auto-detectable settings available with the NTA software (Malvern). The data from the five acquisitions were averaged before determining the distribution and concentration of the particles in each fraction.

### EM

EV samples were subjected to standard negative-stain EM. Briefly, a 5 µL of EV sample was placed on a 400-mesh carbon coated copper grid (Electron Microscopy Sciences) that was glow discharged for 20 s. EV samples were allowed to settle on grids for 5 min in a covered glass dish. Each grid was then quickly washed on 2 drops deionized water, wicked with filter paper, and then stained with 1% PTA for 20 s before wicking dry again with filter paper. A phosphotungstic acid staining technique was used to stain and visualize a number of EV preparations from irradiated and non-irradiated MSC. Twelve grids were prepared per condition, and imaged by an operator who was blinded to sample-treatments; images were counted by trained observers in a blinded, random sequence. Images captured using a JEOL JEM-1400 Transmission Electron Microscope (Tokyo, Japan) equipped with a Gata US1000 CCD camera (Pleasanton, CA). For Immunogold labelling, primary human-reactive mouse anti-CD63 (Abcam) and mouse anti-CD81 (Santa Cruz Biotechnology) were used at 10 µg/mL. Colloidal gold (6 nm) conjugated goat anti-mouse secondary antibody was diluted in buffer at 1:20. Negative controls of RPMI and FBS showed no evidence of nonspecific binding.

### PBL mononuclear cell isolation

Isolation of PBL mononuclear cells (PBMC) was performed as previously described [27]. Briefly, PBMC were separated from freshly collected PBL samples by Ficoll-Hypaque (GE Healthcare) or Lymphocyte Separation Medium (LSM; Cellgro/Corning) density-gradient centrifugation where PBS (Ca^2+^Mg^2+^free; Cellgro/Corning)-diluted PBL samples (PBL:PBS = 1:1) were carefully applied, then centrifuged no brake at 800 × g for 20 min at room temperature (RT). The light-weight layer was then gently collected and washed in PBS and RPMI 1640 (with phenol-red and L-Glutamine; Cellgro/Corning). Cells were then washed twice with RPMI 1640 and the resultant unfractionated PBMC were thereafter manually counted. Cells were then resuspended in R10 medium, which was made from RPMI 1640 (with phenol-red and L-Glutamine; Cellgro/Corning) completed with 10% FBS (Sigma/Atlanta Biologicals) and 1% Antibiotic-Antimycotic [e.g. 100 U/mL penicillin, 100 µg/mL streptomycin, and 0.25 µg/mL Fungizone (amphotericin B); Thermo Fisher]. T cells and monocytes were magnetically removed by immune magnetic cell selection (magnetic-activated cell sorting; MACS) using conjugated magnetic microbeads targeting T cell lineage cell surface markers CD3 and CD14 on LS columns and in MACS buffer (Miltenyi), according to the instructions of the manufacturer. The flow-through T cell depleted PBMC fractions were disaggregated using sterile 35μm filtration (Corning). The negatively selected cellular fractions were enriched for B cells and ASC.

### Fluorescence-activated cell sorting (FACS)

Fresh negatively selected CD3 and CD14 fractions by Miltenyi according to manufacturer’s instruction. Initially the cells were blocked with nonspecific staining by incubating cells with 5% normal mouse serum (NMS; Jackson ImmunoResearch) in PBS for 10 min at RT. Cells were washed and stained with human CD3-PE-Cy5.5, human CD14-PE-Cy5.5 (LifeTech); human CD19-PE-Cy7, human IgD-FITC, human CD27-APC-eFluor780, human CD38-v450, and human CD138-APC (BD Biosciences). After washing, blood ASC (CD19^+^CD27^hi^CD38^hi^) were sorted on the FACSAria II sorter (BD Biosciences). The ASC populations were generally ~90–95% pure. Post-sort ASC were cultured immediately.

### In vitro cultures for human blood ASC

To study ASC survival and IgG-secreting function *ex vivo*, we used cell-free BM-MSC secretome. These cultures were performed on 96-well flat-bottom cell culture plates (Nunc/Corning), maintained in ~150–200uL medium per well, and were set up at 37°C in a humid, 5% CO2 incubator. ASC numbers for each culture well varied (~500 to ~2,036 cells), dependent upon the total post-sort cells from clinical PBL samples. Replicate ASC cultures were maintained without replenishing the BM-MSC secretome or conventional media or vehicle (R10). After days 1, 3, and 6 or 7, each culture was washed 4–6 times to remove secreted Ig and ASC were plated in Elispot wells. The peak percentage of viable ASC during the entire culture (usually on days 1–3) served as 100% survival.

### Cultures with cleanascite

Freshly sort-purified blood ASC populations were cultured in the irradiated (and non-irradiated) BM-MSC secretomes that was pretreated with Cleanascite (CLN), a lipid removal reagent (Biotech Support Group), according to the manufacturer’s recommendations. Similar experiments were also conducted using EV fractions derived from irradiated and non-irradiated secretomes. Secretomes and conventional media (R10) or PBS were used as controls.

### IgG elispot assay

Elispot assays for total IgG were performed as previously described [53]. Briefly, pre-wetted membrane, MultiScreen flat-bottom 96-well ELISpot plates (Millipore) were coated overnight at 4°C with goat anti-human IgG capture *Ab* (5 μg/mL) or with 2 mg/mL BSA (2% in PBS). To prevent nonspecific binding, plates were then blocked with RPMI 1640 supplemented with 8% FBS for 2 h at 37°C. Subsequently, plates were loaded with cultured blood ASC and were incubated in ~150-200 uL media for ~16–18 h at 37°C in the air incubator (5% CO2). Then cells were removed and the plates were washed six times with washing buffer using Microplate Washer (Biotek). Secondary goat anti-human IgG alkaline phosphatase-conjugated *Ab* (1 μg/mL, diluted in PBST + 2% BSA), was then added and incubated for ~2 h at RT. Spots were developed and visualized with an enzymatic color reaction using ABC-AP Vector Blue Substrate reagents (Vector Laboratories). Plates were counted on the ELISpot reader (Cellular Technology Limited; CTL) using the ImmunoSpot 5.0.9.21 software.

### Western blot analysis

Samples (secretomes, EV fractions and HeLa whole cells) were lysed with RIPA buffer and protease inhibitor cocktail or with Mammalian Cell & Tissue Extraction Kit (BioVision), per manufacturers’ instructions. Lysates were resuspended in protein loading dye (Laemmli sample buffer; Bio-Rad) with freshly added β-mercaptoethanol (10%; v/v; Sigma). Sample mixtures were subsequently boiled for 10 min at 90°C and run on 4–15% TGX stain-free precast gels (Bio-Rad). Proteins were transferred to nitrocellulose Immun-Blot® Low Fluorescence PVDF membranes (Bio-Rad) then washed. After blocking, blots were probed with the primary antibodies: anti-TSG-101 (Abcam), anti-ALIX (Millipore; kindly provided by Dr. Shi Hua Li at Emory University), or anti-FN-1 and anti-Cdc42 (Santa Cruz). After incubation, membranes were washed and subsequently probed with IRDye® 680RD goat anti-rabbit or IRDye® 680RD goat anti-mouse (Li-COR), followed by washing. Initial HeLa cells were kindly provided by Dr. Michael Koval (Emory University). Protein concentration was determined using the BCA assay process with Pierce™ BCA Protein Assay Kit (ThermoFisher Scientific), following manufacturer’s instructions. Blots were developed using SuperSignal West Dura Extended Duration Substrate (Thermo-Fisher) and protein bands were detected using the ChemiDoc MP Imaging System (Universal Hood III model; Bio-Rad).

### EV lysis and protein digestion

EV pellets were lysed through end-to-end rotation at 4ºC for 45 min in RIPA buffer. The supernatant was transferred to new tubes. Proteins were reduced with 5 mM dithiothreitol (DTT) (at 56ºC for 30 min) and alkylated with 14 mM iodoacetamide (at RT for 15 min, in the dark). Detergent was removed by the methanol-chloroform protein precipitation method. Purified proteins were digested with 10 ng/μL Lys-C (Wako) in 50 mM HEPES pH 8.6, 1.6 M urea, 5% ACN at 31ºC for 16 h, then with 8 ng/uL trypsin (Promega) at 37ºC for 4 h.

### Peptide purification and LC-MS/MS analysis

A total of 0.5 ug of total protein per sample were used for proteomic analysis. Protein digestions were quenched by addition of trifluoroacetic acid (TFA) to a final concentration of 0.1%, followed by centrifugation to remove the precipitate. The peptides were desalted using a tC18 Sep-Pak cartridge (Waters) and lyophilized and subjected to LC-MS/MS analysis. Peptides were detected with a data-dependent Top20 method [55] in a hybrid dual-cell quadrupole linear ion trap – Orbitrap mass spectrometer (LTQ Orbitrap Elite; ThermoFisher; with Xcalibur 3.0.63 software). One full MS scan (resolution: 60,000) was performed in the Orbitrap at 10E6 AGC target for each cycle, and up to 20 MS/MS in the LTQ for the most intense ions were recorded. These sequenced ions were excluded from further analysis for 90 s. Precursor ions were required to have at least two charges for analysis. Maximum ion accumulation duration was 1000 ms for each full MS scan and 50 ms for MS/MS scans. All MS^2^ spectra were searched using the SEQUEST algorithm (version 28) [56]. Spectra were matched against a database containing sequences of all proteins in the UniProt Human (*Homo sapiens*) database. We used the following parameters for database searching: 20 ppm precursor mass tolerance; fully digested with trypsin; up to three missed cleavages; fixed modification: carbamidomethylation of cysteine (+57.0214); variable modifications: oxidation of methionine (+15.9949). False discovery rates (FDRs) of peptide and protein identifications were evaluated and controlled to less than 1% by the target-decoy method [57] through linear discriminant analysis (LDA) [58]. Peptides fewer than seven amino acid residues in length were deleted. We also applied a filter at the protein level to ensure the protein FDR is less than 1%.

### Bioinformatics

Protein digestion, proteomic analysis and thresholding were performed as described in Methods section, and complete protein lists were generated with the Partek Genomics Suite software (Partek). Full protein lists may be found in Supplemental Table 1. As we have observed ASC to quickly die when grown in vehicle alone, we hypothesized that the additive presence of factors (rather than the absence of apoptotic factors) might provide a better model for ASC survival. In preparing the input dataset for pathway analysis, we included proteins that were differentially expressed in EVs after excluding proteins found in the R10 vehicle alone. Relative abundance was computed as the ratio of the number of hits of a given protein divided by the total number of hits in that sample. Supplemental Table 1 presents these calculations and the paired datasets that were used as input for Partek (which eliminated duplicates and overlapping regions) and then for IPA. The non-irradiated BM-MSC-derived EV fractions afforded 611 such proteins and the irradiated EV fractions afforded a list of 682 proteins. The overlapping region of both the EV fractions (excluding the vehicle alone) was equal to 419 proteins. A standard IPA core analysis was performed using canonical pathways, and full results may be found in Supplemental Table 2. IPA considers the presence of proteins and computes a *p*-value that connotes the likelihood that a predefined molecular biology pathway has been activated. Within this software package, a statistically significant *p*-value of 0.05 is equivalent to a – log(*p*-value) = 1.35.

### Statistics

Graphical data for the project was analyzed using GraphPad Prism versions 6.0 and 7.0. Figures 1 and 4 were analyzed via two-way ANOVA. For Figure 2(c,d), microscopy staff were blinded to treatment status of preparations; after image collection, two lab technicians were trained on how to identify vesicular bodies in the digital image files, again in a treatment-blinded fashion, using a mouse cursor scaled 50–100 nm. HPFs from 12 grids were thus enumerated. Due to the counted, integer-based nature of these data, a Mann–Whitney test was used to assess significance.
